# Hospital physicians’ reasoning and information needs in the diagnosis and prevention of drug-induced acute kidney injury: a qualitative study

**DOI:** 10.1186/s12882-026-04852-x

**Published:** 2026-03-04

**Authors:** Joris E. Lieverse, Kyrill N. A. Visser, Linda Dusseljee-Peute, Joanna E. Klopotowska, Stephanie Medlock

**Affiliations:** 1 Department of Medical Informatics, Amsterdam UMC location University of Amsterdam, Meibergdreef 15, Amsterdam, 1105 AZ The Netherlands; 2 Amsterdam Public Health, Digital Health, Amsterdam, The Netherlands; 3 Amsterdam Public Health, Quality of Care, Amsterdam, The Netherlands; 4 Department of Medical Informatics, eHealth Living & Learning Lab, Amsterdam UMC Location University of Amsterdam, Meibergdreef 9, Amsterdam, the Netherlands; 5 Amsterdam Public Health, Aging and Later Life, Amsterdam, The Netherlands; 6 Amsterdam Public Health, Methodology, Amsterdam, The Netherlands

**Keywords:** Decision support, Diagnostic reasoning, Drug-induced acute kidney injury, Hospital physicians, Information needs

## Abstract

**Background:**

Acute kidney injury (AKI) is a common and severe complication in hospitalized patients. Nephrotoxic drugs significantly contribute to AKI. Diagnosis and prevention of drug-induced AKI (d-AKI) could be improved, but little is known about its occurrence despite preventive efforts. Therefore, the objective of this study was to investigate hospital physicians’ reasoning about d-AKI and gain insight into the challenges they encounter, including identifying unmet information needs surrounding d-AKI.

**Methods:**

We conducted semi-structured interviews with hospital physicians from diverse specialties and experience levels at one Dutch academic hospital. Two interviews were independently open coded by 3 researchers and the resulting codes/themes organized by consensus. Using the resulting code tree, we conducted thematic analysis of the interviews.

**Results:**

Thirteen interviews were conducted (6 women, 7 men), with physicians’ professional experience ranging from 3 to 36 years. The group comprised 9 specialists and 4 residents. For many of these physicians, managing AKI takes priority over diagnosing d-AKI. AKI cases with multifactorial etiology and limited d-AKI awareness were mentioned as reasons for delay in or lack of d-AKI diagnosis. Physicians emphasized monitoring and risk assessment with regards to d-AKI prevention. While kidney function is generally well-monitored during hospitalization, there are circumstances where the urgency of treatment may result in less weight on nephrotoxic potential. Physicians expressed the need for an up-to-date home medication overview, monitoring guidance around nephrotoxic drugs, and better prediction of d-AKI.

**Conclusion:**

This study highlights key aspects of current hospital practices surrounding d-AKI. Diagnosing d-AKI often has a low priority, which may explain why it is often underdiagnosed and not registered. Prevention is limited by gaps in monitoring and the need for nephrotoxic drugs in urgent situations. We emphasize the need for improved d-AKI diagnosis and clinical awareness, and providing personalized d-AKI prevention strategies.

**Clinical trial number:**

Not applicable.

**Supplementary Information:**

The online version contains supplementary material available at 10.1186/s12882-026-04852-x.

## Background

Acute kidney injury (AKI) is a frequent clinical complication occurring in approximately 20% of hospitalized patients [1]. Hospital-acquired AKI is associated with a 4- to 10-fold increase in mortality and substantially higher costs [2, 3]. The majority of hospital-acquired AKI cases are attributed to prerenal AKI, (e.g., caused by reduced renal perfusion); acute tubular necrosis (ATN) involving ischemic or toxic injury to tubular epithelial cells; or acute tubulointerstitial nephritis (TIN), an inflammatory injury of the tubules and surrounding interstitium that impairs kidney function. The pathophysiology of AKI is often a result of multiple interacting factors, with drugs accounting for approximately 14–37% of AKI cases [4–7]. To prevent drug-induced AKI (d-AKI), physicians need information on known-nephrotoxic drugs, but this information is scattered across multiple, sometimes-conflicting sources [8, 9]. They also need to be able to monitor renal function and take preventative measures, but the evidence base is thin for both dosing recommendations and outcomes in high-risk patients such as those with chronic kidney disease (CKD) [10, 11]. 

In practice, we know that d-AKI often goes unrecognized [12, 13], even though this can lead to severe complications, such as the need for dialysis [14]. Many cases of d-AKI are preventable [15]. However, little is known about when and why d-AKI occurs despite preventative efforts. Renal function monitoring guidelines exist for many nephrotoxic drugs, yet gaps in monitoring still occur, with little insight as to why [16]. 

Few studies have investigated hospital physicians’ perspectives on drug management in renal diseases [17], and to the best of our knowledge, physician perspectives on the detection and prevention of d-AKI have not been studied. This gap limits our ability to understand the aforementioned problems, and propose solutions. It is likely that a lack of readily-accessible information contributes to these problems. Clinical decision support systems (CDSS) can help [18–20], but we first need to understand when clinicians would use such resources and where they experience a need for information and support. Previous studies have used qualitative methods for capturing the perspectives and needs of healthcare professionals, in areas such as CKD [21, 22], AKI [23], and prescribing errors [24]. 

Therefore, the aim of this study is to investigate how hospital physicians reason about d-AKI and gain insight into the challenges they encounter, including identifying unmet information needs surrounding d-AKI.

## Methods

The Consolidated Criteria for Reporting Qualitative Health Research (COREQ) checklist was used [25]. This study is part of the LEveraging real-world dAta to optimize PharmacotheRapy outcomes in multimOrbid patients by using machine learning and knowledGe representation methods (LEAPfROG) project [26], which aims to develop a data-driven medication safety system to improve d-AKI detection in CKD patients.

### Study design

**Fig. 1:**
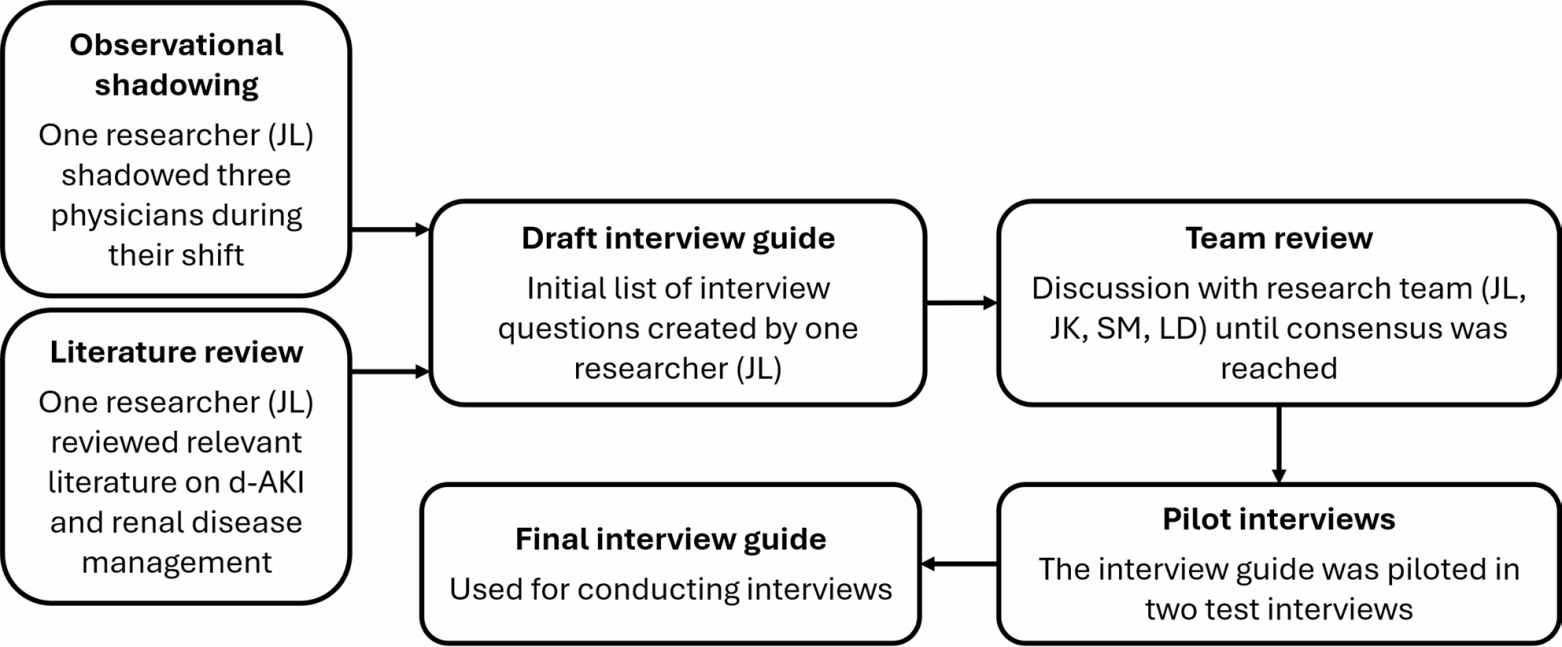
Development of interview guide. The literature used to develop the interview guide is listed in the appendix. Physicians who were shadowed or who participated in the pilot interviews were excluded from participation in the study

**Fig. 2:**
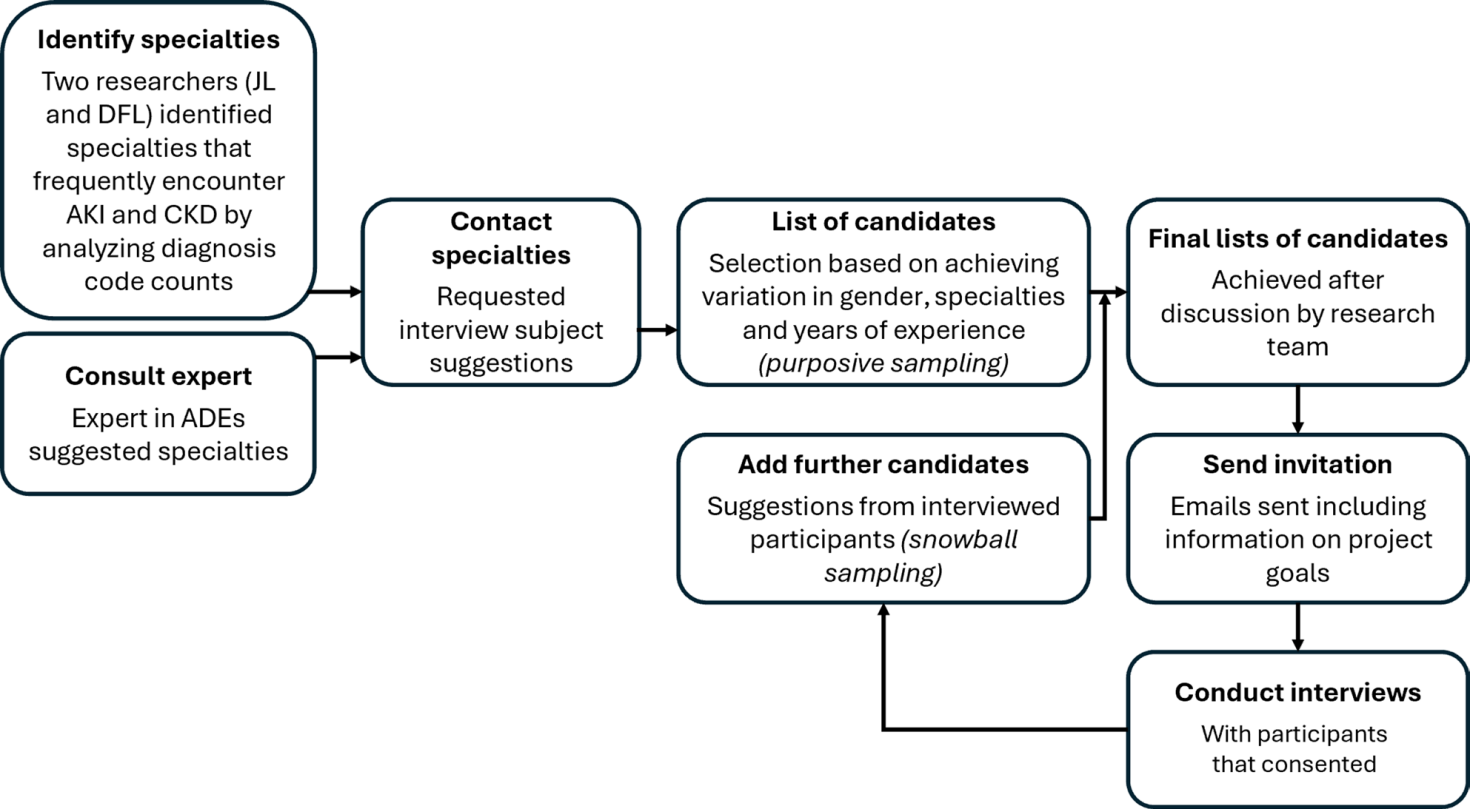
Participant recruitment and inclusion. During the course of the interviews, additional participants were added to the list of candidates

Between September 2023 and January 2024, we conducted semi-structured interviews with hospital physicians in a large academic hospital in the Netherlands. The Medical Ethics Committee provided a waiver from formal approval (W22_340 #22.412) since the LEAPfROG project, including all sub studies, does not fall within the scope of the Dutch Medical Research Involving Human Subjects Act. Figure 1 outlines the process that led to the creation of the interview guide, Fig. 2 depicts the recruitment and inclusion of participants. Data collection concluded upon reaching saturation, defined as three consecutive interviews with no new major themes.

### Data collection

Interviews were performed by one researcher (JL), a pharmacist and PhD student in Medical Informatics. Interviews were conducted in Dutch, and recorded using Microsoft Teams [27]. Transcripts were generated automatically and corrected by two researchers (JL and KV). Informed consent was obtained from all participants.

### Coding and thematic analysis

Two interviews were selected at random to be independently coded by three researchers (JL, SM, LD) using an open coding approach. In open coding, the researchers create codes which summarize the ideas voiced by the interview participants. The goal is to accurately reflect the topics that are important to the participants. Codes were discussed to reach consensus, and an initial code tree generated [28]. Two researchers (JL and KV) independently coded the remaining interviews using MAXQDA2022 [29], with disagreements resolved by discussion. If no agreement was reached, a third researcher (SM) made the final decision. Final main and sub-themes were established through discussions among the researchers (JL, JK, SM, LD, KV).

## Results

### Interviews

The interview guide and code tree can be found in the supplemental materials (Table S1, Table S2). A total of 35 physicians were invited, of which 16 agreed to participate. We conducted 13 thirty-minute interviews with 6 male and 7 female physicians. The group comprised of 9 specialists and 4 residents (4 cardiologists, 3 nephrologists, 3 internists (2 geriatric and 1 general), 1 surgeon, 1 intensivist, and 1 general hospitalist), with 3 to 36 years of experience.

The following section is organized by the three interview topics. In keeping with the goal of accurately reflecting the views of the participants, the results under each topic are organized by the themes that emerged from analysis. Supporting quotes are provided in Table 1.

### Interview topic: D-AKI diagnosis

#### Reasoning

A majority of physicians (11/13) indicated that diagnosis of d-AKI is mainly a process of determining whether a drug is the most likely cause of an AKI. All physicians (13/13) said that suspicion of d-AKI is increased when the patient is taking a drug that is known to be nephrotoxic. Almost half of the physicians (6/13) gave examples of multifactorial AKI, noting that kidney impairment often reflects an interplay between drug and disease effects. One physician indicated that d-AKI is often considered only when renal function does not recover as expected.

#### Timing

Physicians had different opinions on the time to AKI diagnosis. Of those who elaborated on this topic (9/13), four stated that an AKI should be classified as pre-renal, renal, or post-renal within 24 h; the others (5/9) indicated that it can take much longer (3–7 days). In outpatient settings, evaluation often occurs 1–3 weeks post-discharge. In the inpatient setting, initial AKI assessment occurs within hours of admission but it takes several days to assess other possible causes. The intensivist mentioned that d-AKI may remain undetected until patients have improved and been transferred out of the intensive care. Almost all physicians (11/13) mentioned considering the timing of drug exposure relative to the onset of AKI when establishing causality.

#### Laboratory measurements

Almost all physicians (12/13) pointed out the importance of laboratory tests, particularly serum creatinine (SCr), although six mentioned its limitations: e.g., that it is not specific for AKI, and that it is suboptimal for informing about kidney function. All physicians in our interviews said that they follow trends in SCr, not just absolute values. Six physicians mentioned that drug concentration levels in blood (i.e., therapeutic drug monitoring; TDM) take precedence over SCr levels if available.

Seven physicians mentioned that urine sediment examination is informative, although two cardiologists said that it is more suited for internists or nephrologists. Most physicians (7/13) also mentioned the use of (hourly) urine output, although one pointed out that it is not routinely recorded.

#### Other diagnostic measures

Four physicians (nephrologists or internists) mentioned the use of kidney biopsies to find concrete d-AKI evidence. Four physicians indicated that this procedure is invasive and rarely done, making confirmation of d-AKI difficult to achieve. Three physicians mentioned ultrasound as useful for ruling out post-renal causes.

#### Clinical awareness of d-AKI

All (13/13) physicians indicated that they are aware of the potential for AKI in drugs that they prescribe regularly. However, four were less comfortable with assessing AKI potential in drugs that they rarely prescribe; two mentioned use of knowledge bases to check this. Four physicians (nephrology and internal medicine) reported that they believe other specialties are less aware of the nephrotoxic potential of drugs. One nephrologist and one internist explicitly stated that when assessing AKI they would always consider d-AKI as a possibility. Three physicians noted that nephrologists or internists are more likely to diagnose d-AKI.

#### Margin of uncertainty

Four physicians mentioned identifying ‘why not’ scenarios (e.g., why did the patient *not* urinate, or why is kidney function *not* improving as expected) as part of (d-)AKI suspicion. When d-AKI is suspected, most physicians (10/13) mentioned stopping suspected drugs and a few mentioned adjusting dosage (2/13). Two physicians mentioned that d-AKI is diagnosed by observing the kidney function after discontinuation or dose adjustment, although one mentioned that discontinuation is not always feasible.

Most physicians (10/13) indicated that they view the diagnosis of d-AKI as a shifting of probabilities. A majority (12/13) aim to establish the most likely AKI etiology, but almost never prove it; nor is it perceived as a high priority to them. Seven physicians mentioned the lack of objective diagnostic evidence in d-AKI. Four others mentioned the constant challenge of ruling out other causes.

#### Acceptable decline in kidney function

All physicians (13/13) stated that they accept some degree of estimated glomerular filtration rate (eGFR) loss for some drugs, and a majority (9/13) stated that with some specific drugs (e.g., angiotensin-converting enzyme (ACE) inhibitors) a drop in kidney function is expected and accepted. Three physicians stressed the importance of avoiding permanent, irreversible kidney damage and three mentioned that temporary and reversible kidney function decline is tolerated for these drugs.

### Interview topic: D-AKI prevention

#### Laboratory measurements

Almost all physicians (12/13) mentioned laboratory monitoring (e.g., SCr, TDM) as a critical component for d-AKI prevention. However, one cardiologist noted that it can be unclear whether an increase in SCr warrants action.

#### Risk assessment

Almost all physicians (11/13) indicated that potentially nephrotoxic drugs are avoided or used more cautiously in high-risk patients, with more monitoring and a lower threshold for intervention.

#### Dilemmas

A majority (8/13) of physicians acknowledged the challenge of balancing the need to effectively treat a condition with the potential risk of kidney dysfunction and/or damage. Three physicians mentioned that more risk-taking might also be necessary in high-risk patients. Two physicians described situations where it was unclear whether their actions were beneficial or harmful. Another emphasized the challenge of distinguishing between a drug’s intended mechanism of action and its side effects. One physician noted that it is important to consider whether cases of d-AKI are avoidable, but that 100% perfection is unlikely to be achieved.

#### Clinical awareness of d-AKI

Two physicians mentioned that d-AKI (suspicion) should be thoroughly documented in the patient’s medical history to help prevent recurrent d-AKI due to the same drug. Four physicians mentioned training and protocols as important preventive tools whereas others (4/13) mentioned knowledge of which drugs are potentially nephrotoxic. According to most physicians (7/13), understanding how a drug affects the kidneys and continuously monitoring its impact is essential.

Four physicians (including two nephrologists and one internist) noted that raising clinical awareness would aid in prevention, especially in non-internal-medicine departments. The interviewed surgeon indicated surgeons have less awareness of d-AKI because patients are usually transferred before d-AKI is detected.

#### D-AKI despite preventive measures

All physicians mentioned reasons why d-AKI occurs despite preventive efforts. Almost all (10/13) stressed how in acute or critical situations, the priority may shift toward using effective – but potentially nephrotoxic – drugs. Five physicians noted that at times, there are no safer alternatives. Six physicians mentioned gaps in renal function monitoring as another important reason, for example due to a break in communication with the patient or other providers, or (for inpatients) the physician’s attention being fragmented between multiple patients.

Inadequate patient instructions were also mentioned by six physicians as a risk for outpatients, for example the instruction to avoid over-the-counter (OTC) non-steroidal anti-inflammatory drugs (NSAIDs).

### Interview topic: Information needs around d-AKI

#### Missing and unavailable data

Almost half of the physicians (6/13) indicated that they cannot rely on the accuracy and completeness of the home medication list in the patient record. They cited multiple conflicting medication lists, OTC drug use and discrepancies between use and documentation. Some physicians (5/13) mentioned the need for an up-to-date and accurate home medication overview. In contrast, one physician affiliated with the surgical oncology department said their home medication list is very up-to-date, because medication checks are part of their protocols.

#### Information needs related to nephrotoxic drugs

Some physicians (4/13) also expressed a desire for better information about nephrotoxic drugs and interactions, including guidance on monitoring. Three internal medicine physicians mentioned that TIN is hard to prevent, because of the lack of knowledge regarding TIN and low sensitivity of SCr. TIN mediated by allergic reactions is less recognized and less known, according to another internist. Another nephrologist stated that non-nephrologists identify TIN less frequently, due to its subtle manifestation combined with diagnostic limitations related to SCr.

#### Information needs related to CDSS

Regarding CDSS, two mentioned that medication dosing alerts related to renal function are based on absolute SCr values, while relative changes or trends might be more clinically appropriate. Four physicians proposed automation that enables scheduling and tracking of SCr checks. Five physicians suggested that a tool for calculating the likelihood of d-AKI, including known AKI risk factors, would be useful. Some suggested alerts recommending intervention by a nephrologist or pharmacist (5/13). However, a majority (7/13) mentioned alert overload, or warn for such consequences in future systems.

**Table 1:** Illustrative quotations from interviews with physicians

Themes related to d-AKI diagnosis	Quotes
Reasoning	The subtle relationships are, of course, much harder to figure out. If somebody had a very high dose of gentamicin, then you’re almost sure that that was the cause. Combined with ruling out other things. But sometimes we don’t know exactly, do we? Someone who also had a bit of low blood pressure, well then of course it is difficult to say with certainty that it is really medication-related. *(Nephrologist)*
Timing	What you often see is: someone comes in, in shock. Well, the kidney takes a hit as well on day zero. … Day two may be the peak [in creatinine], because it takes a while before you see it in the blood. After that you expect it to go down again, because if all goes well, the patient has stabilized a little bit. And I think only if it continues to rise, then d-AKI starts to enter our thought process. Obviously, you would prefer to have that much sooner. It’s just that it takes a long time before you see the effect in your [creatinine]. With renal function it just takes a while, and that itself is difficult. *(Intensivist)*
Laboratory measurements	Maybe what we should do more often is simply collect 24 h’ of urine, to get more information about the renal function. *(Intensivist)* Of course you look at the [creatinine]. We use that as a measure of renal function, even though that isn’t necessarily the best measure. We know, for example, that with ACE inhibitors – which we use a lot around here – you can accept a rise in [creatinine], without it necessarily being a deterioration of the kidney function. That’s also true for cotrimoxazole. That can also cause a rise in creatinine without any real renal function loss. That makes it all pretty complex. *(Intensivist)*
Other diagnostic measures	I think the difficulty is simply that we never have hard evidence, unless of course you take a biopsy, but we generally prefer not to do that. *(Nephrologist)*
D-AKI awareness	[Q: How do you know which medications pose a high risk of AKI? ] For some medications, you just know it. They are infamous. *(Internist geriatrics)* As a nephrologist, any time there is an AKI, you consider that it could be a d-AKI. *(Nephrologist)*
Margin of uncertainty	Naturally, we admit a lot of patients with heart failure. The people that are treated with diuretics sometimes get a worsening of renal function. Is that then medication-induced or is it simply someone who has progressive heart failure that is now less hydrated? Naturally, that is sometimes complicated. Is that just the function of the medication, or should you say that it’s drug-induced? *(Internist geriatrics)* [Q: How difficult is it to really confirm that there is a causal relationship between the drug and AKI? ] If you have the ability to stop [the medication] and it is reversible, then you’ve got it, but I think that is exactly the crux. I think you look when the renal function decline occurred, and which medications or combination of medication preceded it. I think mainly about the timeline, but there is always a differential diagnosis, isn’t there? Because you gave a medication, but they are also hypotensive, and on top of that they have an acute tubular necrosis. Yeah, what is it, then? I think it is certainly a combination [of these causes]. *(Internist geriatrics)*
Acceptable decline in kidney function	[Q: How much renal function loss would you consider acceptable? ] Naturally, that depends on the cause of the AKI. If it is an expected, logical effect associated with the mechanism of action of the drug, if it is reversible after discontinuation without lasting damage, then you can accept quite a bit. But if it’s a TIN (for example), then you know that it’s not going to get better – only worse – if you continue with this medication. Thus then in the long term no loss is acceptable. *(Nephrologist)* I think it varies per medication. If you think that it’s reversible, like for example with ACE inhibitors, then you accept an increase in [creatinine] of 20% [relative to baseline]. But for example with tacrolimus you don’t really look at this much, you titrate based on blood level, and try to keep the blood level as low as possible [Implicitly: within the therapeutic range]. *(Nephrologist)*
**Themes related to d-AKI prevention**	
Laboratory measurements	Again, you look at [creatinine]. That’s what we use as a measure of renal function. *(Intensivist)* What do you have to do when you start a specific medication and how you need to treat it if there is a rise in [creatinine], at what level of [creatinine] rise should you do something? That’s already a bit of guesswork. *(Cardiologist)*
Risk assessment	I think that it is good that you are extra alert with high risk patients that get certain medication, so that you can intervene early – that’s the case with most forms of d-AKI, ultimately, the earlier you see it and the sooner you can change medications, the better the chance that it won’t lead to renal replacement therapy and that things are still largely reversible. *(Nephrologist)*
Dilemmas	There are definitely medications that we know can cause d-AKI, but we still give it, because we know that it’s the best medication. *(Intensivist)* In medicine we have the adage, “Treat first, what kills first.” Thus if someone is septic or had a massive heart attack, then first you have to make sure that the patient survives, with all the medication that is needed to do so. There is a risk that the renal function worsens or maybe even leads to renal failure or severe AKI… yeah, sometimes that’s a risk we must take. *(Nephrologist)*
D-AKI awareness	The protocols are pretty strict, if the renal function is poor. Thus the chance that you see a d-AKI… yeah, now and then you will see one. *(Surgeon)* You can’t create “awareness” everywhere about everything. A surgeon won’t on his own be continuously thinking about d-AKI. That’s not attainable. *(Internist general medicine)*
D-AKI despite preventive measures	I think that with conscientious prescribing, good instructions to the patient about fluids, interactions, old medicines, new medicines and a low-threshold for monitoring of kidney function, you can prevent a large portion of at least the permanent renal damage. Temporary renal damage, not entirely, because that’s what you are doing the monitoring for, so that you can stop it in time. But I’m not the type of doctor that has the illusion that with all our systems we can prevent every form of renal damage. *(Internist)* Look, if you prescribe a new medication, then you get a warning that it can have an interaction with renal function, usually you then think: well, yeah, but this really must be given. *(Intensivist)*
**Themes related to information needs**	
Missing and unavailable data	There can be a substantial difference between what medication we think that someone is using, what the pharmacy has, what the general practitioner has, and thus the communication about medication use is, well, simply a lot of work to figure out and track down… but how to do it better, I don’t know. *(Internist geriatrics)* But it is much more difficult to get an overview of what nephrotoxic medication a patient is getting now, at this moment. *(Intensivist)*
Information needs related to nephrotoxic drugs	I think that other specialisms don’t think about it as much, particularly specific antibiotics or medications that are notorious for it [causing d-AKI], with other specialisms that is much less and you can imagine that it usually goes well, so you don’t see a TIN very often. But we see it quite often, because naturally that’s the moment we get called in. Thus our chance of catching it is higher. Sometimes awareness is also greater, naturally, where other specialties use some medications really often – daily, weekly prescriptions – but naturally only encounter something like this a few times per year. *(Nephrologist)*
Information needs related to CDSS	Look, if you can get better information like “Hey, on the basis of this pattern, this is likely to be caused by this medication that you prescribed a few days ago, or someone else started a few days ago"… that would really help. *(Cardiologist)* For the doctor, artificial intelligence would mean a more personalized risk profile. *(Surgeon)*

## Discussion

In this study we investigated the challenges hospital physicians encounter surrounding d-AKI and identified unmet information needs. In diagnosing d-AKI many of the interviewed physicians aim to establish the most likely cause of the AKI, but often do not prove it. Identifying whether an AKI is a d-AKI is secondary to managing the AKI itself, especially when multiple potential causes exist. Diagnosis of d-AKI may be delayed in complex patients, due to the difficulty in ruling out other causes. In terms of d-AKI prevention, our interviewed physicians underscored the importance of laboratory monitoring. AKI risk assessment is also important, both for deciding whether to use a potentially nephrotoxic drug and how intensively the patient should be monitored. Even so, renal function monitoring is not always done, or not done on time, sometimes leading to d-AKI events. It should be noted that these monitoring gaps are reported as physicians’ experiences rather than as measured outcomes. D-AKI prevention is difficult in acute situations. Physicians prioritize the most-needed care (e.g., treating sepsis) and nephrotoxic drugs may be prescribed despite AKI risk, sometimes leading to reduced focus on kidney safety. We identified several important information needs, including the need for an up-to-date overview of home medications reconciling information from various sources. Physicians would like better information on nephrotoxic drugs, and better support for monitoring. Finally, physicians felt that a more personalized risk profile for d-AKI could help in d-AKI diagnosis and prevention, although they expressed concerns about alert overload. However, this work does not validate these solutions; these results reflect the opinions of our participants.

To our knowledge, this is the first study to investigate the processes and information needs of hospital physicians in d-AKI diagnosis and prevention. A few other studies have investigated information needs of physicians in other related care processes. Phipps et al. highlighted challenges in managing drugs for patients at risk for AKI across care boundaries [17]. Like our study, they noted that incomplete data complicated AKI management. They also noted the limits of renal function biomarkers and the difficulty of balancing AKI management with treatment of other comorbidities. Elkhadragy et al. investigated medication-related decision-making in patients with renal disease [30]. In both this study and ours, participants mentioned that automated alerts are not sufficient and careful monitoring is needed. Likewise, participants mentioned balancing the need for the medication with the risk of renal damage.

The primary focus on treating acute conditions, even at the risk of renal damage, is a critical finding of our work. The situations described by our participants can be divided into two main scenarios: acutely ill patients (e.g., in ICUs) and stable patients in routine care. D-AKI occurs in both groups, but AKI in the latter group is arguably more likely to be iatrogenic, and more preventable. A delicate balance was mentioned in high-risk and critically ill patients with multiple AKI risk factors. Such patients are strictly monitored, but there are limited options for milder therapies and moderate doses. Sometimes, our physicians accept a renal function decline if the drug is needed to treat a problem that is perceived as more urgent or severe. This suggests that clinicians in such a situation might be less inclined to seek information about minimizing renal side effects. The remark about d-AKI not being recognized until after ICU discharge is particularly noteworthy. This may reflect a broader, systems-level issue in critical care. Our participants highlighted two contributing factors: first, the tendency to only consider d-AKI if kidney function fails to improve as expected, and second, the under-recognition of d-AKI in departments with a short average length of stay. Together this implies a high likelihood of missed cases in such situations.

This aspect of clinical practice extends beyond cognitive burden, impacting healthcare systems and workflows at a broader level. This implies a need for workflows that make nephrotoxicity assessment “automatic” rather than an added cognitive burden. Automated causality assessment tools and well-trained, multidisciplinary teams, including clinical pharmacists, could play a role.

Our participating physicians generally approach diagnosing d-AKI by ruling out other causes of an AKI and attempting to establish a causal relationship between AKI and the suspected drug. Causality assessment tools for adverse drug events (ADEs) have been proposed to assess the causal relationship between a drug and adverse event [31–33]. Although the physicians in our study did not explicitly report using formal causality assessment tools, most did mention discontinuing what they considered the offending drugs, and monitoring changes in kidney function. Most referred to timing, but only a few articulated time-to-onset reasoning as applied in formal causality assessment. Although it could be that they consciously perform a dechallenge and consider temporal relationships (but did not explicitly articulate it), this more likely reflects a trial-and-error approach rather than deliberate causal reasoning. Clinicians are prone to cognitive biases such as anchoring and premature closure, as demonstrated in numerous studies on diagnostic error [34]. Anchoring refers to the tendency to adhere rigidly to an initial impression, potentially disregarding subsequent evidence that contradicts or disproves it. Premature closure refers to reaching a conclusion before all relevant or necessary evidence has been adequately considered. These cognitive biases appear to affect physicians regardless of level of experience and may, in fact, be more prevalent among experts. Therefore, tools to support clinicians in causal reasoning around d-AKI may be of value. Furthermore, the physicians in our study implied that drug causality is often not confirmed in AKI cases. This might help explain why d-AKI is often not registered in the patient record [35]. This also has implications for d-AKI pharmacovigilance [12]. If d-AKI is not documented, then there is less chance of detecting a new or changing safety issue. The results also imply that risk management needs to be more proactive. Having domain knowledge resources with drug side-effects (e.g., websites, handbooks) available is not enough; clinicians cannot be expected to know every adverse effect for every drug they may encounter. We need to support clinicians by making the information more accessible at the time of prescribing and during the diagnostic process.

The observation by our participants that nephrologists or internists are more likely to diagnose d-AKI suggests the persistence of expertise-specific silos, which may hinder timely recognition. Considered in terms of clinical decision-making frameworks [36], it seems that clinicians rely partly on clinical intuition (suspicion of d-AKI when the patient is taking a nephrotoxic drug familiar to the clinician) and partly on a Bayesian/threshold approach [37]. Viewing our participants’ responses in this light: Lack of awareness of d-AKI leads to a low estimation of prior probability. The action threshold for stopping the drug may be too high (priority is placed on gathering evidence to rule out other causes) or too low (drugs may be stopped unnecessarily without explicit evaluation of causality). Although some of the barriers to d-AKI prevention are difficult to address, others suggest improvements to the design of electronic decision support or automated diagnostic aids. In the LEAPfROG project, we are developing a digital causality assessment tool to help physicians determine drug causality in cases with multiple possible causes of AKI. We are also developing a personalized AKI risk prediction model to help identify and call attention to high-risk patients, as well as a patient-specific tool for checking side effects in patients with polypharmacy (including drug-drug interactions). Such tools must be designed to fit with physicians’ mental models of AKI as multifactorial and d-AKI as a “probable” rather than “definitive” diagnosis. Medication alerts in our hospital recommend adjusting dosage in response to specific SCr values, or starting TDM in response to specific values of SCr. These SCr-based alerts do not take into account SCr trend over time and lack personalization. More personalized alerts have reduced d-AKI in children; this may also be useful in adults [38]. Other issues may be addressable as new information becomes available, such as patient-specific advice on what degree of eGFR decrease should be accepted with certain medications. In all cases, CDSS must be designed to fit with the workflow and provide actionable recommendations (e.g., “Schedule follow-up SCr in 48 hours”). CDSS should not be limited to alerts, but should provide support in other ways (e.g., risk stratification).

The physicians pointed out the limitations of SCr measurements for timely d-AKI detection. Apart from the biomarkers mentioned in the interviews, such as SCr and urine sediment exam, other (novel) d-AKI biomarkers which have been proposed to differentiate d-AKI from other etiologies, such as serum Cystatin C, Proenkephalin, Neutrophil Gelatinase-Associated Lipocalin, Urine chemokine C-X-C motif ligand 9, and Urine beta-2-microglobulin [39]. However, these biomarkers are rarely used in current clinical practice and biomarker research in AKI is not yet complete [40]. At our institution, the more established biomarker, serum Cystatin C, is reserved for select cases due to its substantially higher assay costs compared with SCr. Many of the physicians seem to rely on the kidney’s natural reserve capacity when prescribing certain drugs like ACE inhibitors. This probably reflects mechanism-dependent reasoning, guiding whether a drop in kidney function is interpreted as acceptable (i.e., predictable hemodynamic effects vs. possible tubular injury). However, the apparent conceptual overlap between hemodynamic effects and injury could contribute to diagnostic uncertainty and influences risk–benefit assessments when evaluating kidney function decline. This could also lead to overconfidence in AKI reversibility and a delay in stopping the responsible agents. For example, in patients with volume depletion or renal artery stenosis, the decrease in GFR may be significant [41]. AKI can occur due to superimposed injury or concurrent administration of other drugs with renal effects. Moreover, defining an appropriate threshold for stopping or adjusting the dose of these drugs remains a challenge [42]. Several physicians also singled out drugs linked to TIN, like proton pump inhibitors (PPIs) [43, 44]. Studies show that PPI use is associated with higher risks of CKD and AKI, implying a high likelihood of unrecognized drug-induced TIN [45, 46]. The idiosyncratic nature of TIN makes it difficult to prevent and, often, difficult to diagnose. A combination of suspect drugs and other data can raise the index of suspicion. Signs of an allergic reaction may point to TIN. Eosinophiluria has been associated with TIN [44], but its low sensitivity and specificity limit its utility [47, 48]. Beyond the role of novel biomarkers, management of TIN could benefit from early alerts targeting at-risk patients who are susceptible to subtler kidney function decline. A recent dysfunction/damage model was proposed that could be useful to guide clinicians in reasoning about differential mechanisms of d-AKI [49]. 

Half of the physicians specifically mentioned the lack of an accurate home medication list as a barrier. This is consistent with reports about frequent unintended discrepancies between patients’ medication lists across transitions in care, including OTC drugs [50]. It also echoes the findings of Mastellos et al. regarding information needs of general practitioners, who also expressed needs regarding communication and synchronization of data between care settings, including medication lists [51]. However, our findings suggest a broader problem with reconciling home medication data from disparate sources, as well as the accuracy and timeliness of this data. This can lead to delays in assessment of AKI causes. It should be clarified that the perceived inaccuracies in home medication lists reflect physicians’ perceptions, as we have not verified these results against actual medication list data.

A strength of our study was the use of a qualitative approach, allowing participants to share their experiences with AKI and d-AKI within a broad clinical context. All interviews were coded independently by at least two researchers, and the initial code tree established through coding by three researchers. Our choice of physicians to interview was data- and expert driven, and “snowballing” was applied in addition, leading to a diverse sample.

Our study also has limitations. Although our sample was diverse, this is a single-center study. We expect that physicians in other settings encounter similar problems, but performing a similar study in a new setting could check whether this is the case. Our recruitment strategy may have introduced a selection or conformity bias. However, contacts of the researchers were asked to help us identify interview subjects, thus most subjects were not known to any of the researchers. Physicians with an interest in d-AKI are probably more inclined to participate, thus a participation bias is also possible. Although we believe the combination of data-driven and snowball sampling resulted in a good sample of participants, it is possible that inclusion of additional specialties (such as oncology or emergency medicine) would yield additional insights. Although we reached saturation in our major themes, our sample included only one representative from some important groups, such as intensivists and surgeons. This may result in limited examples and depth in the perspectives of these groups. We chose to allow the interview participants to form their own interpretation of what is encompassed by “AKI” and “d-AKI”, to gain insight into their conceptualization of the topic. This approach may have led to greater variability in the responses; for example, from those working in critical or noncritical care settings. Finally, all interviews were conducted by a single researcher. While potential bias from the interviewer’s background and unconscious variation in follow-up questioning cannot be fully excluded, using a team-developed interview guide and independent coding enhanced methodological rigor.

We included both specialists and residents in our sample, but our study was not designed to detect differences between them. Future studies could use a quantitative approach to investigate this question, and the results used to inform training. Likewise, our interviews were broad in scope and did not go into depth on topics such as differential mechanisms of drug interference; future studies could investigate this in more detail. Although our participants spoke mainly about AKI in a hospital population, it is not only a “hospital problem.” D-AKI can be caused by medication prescribed outside the hospital, or present in other settings. However, because kidney function is frequently measured during hospital stay, AKI is more likely to be detected at an earlier stage compared with settings where measurements are irregular or performed only on demand, such as in general practice. This makes the prevention and diagnosis of d-AKI even more challenging, necessitating alternative strategies. Both the interviewed physicians and previous work [52] noted problems at care transitions, and future work could look at GPs’ perspectives on this issue. Likewise, the important role of hospital pharmacists was mentioned and bears further investigation [53]. The findings of the present study are also expected to inform future work in the LEAPfROG project and research on the topic of information needs surrounding d-AKI on a broader scale.

## Conclusions

This study highlights effective aspects of current practice and identifies areas for improvement in the clinical management of d-AKI. The physicians’ primary focus is on treating acute conditions which draws focus away from diagnosing and preventing d-AKI. D-AKI diagnosis is often delayed, especially in complex patients, and often never confirmed. Physicians consider the elements of formal causality assessment, but do not seem to follow a standardized diagnostic pathway. Better pharmacotherapeutic guidance is needed in order to interpret where acceptable renal function decline ends and damage starts. Our findings suggest the need for improved diagnostic methods, better data quality, increased clinical awareness, and a more personalized approach to d-AKI prevention. This highlights the potential value of integrating risk-based alert systems into clinical workflows to improve AKI-related decision-making. Clinical decision support systems could aid physicians in registering d-AKI and identifying and understanding the relationship between drugs and AKI, thereby improving kidney safety.

## Supplementary Information

Below is the link to the electronic supplementary material.

Supplementary Material 1: Table S1: Interview guide. Table S2: Code tree

## Data Availability

The data collected during this study are part of the larger LEAPFROG project and contain privacy-sensitive information. In accordance with the conditions outlined in the ethical waiver, we do not have permission to share the data.

## Declarations

### Consortium Members

Ameen Abu-Hanna^7^, Annette ten Teije^8^, Birgit A. Damoiseaux-Volman^7^, Cornelis Boersma^9^, Dave A. Dongelmans^10^, David H. de Koning^7^, Frank van Harmelen^8^, Gerty Holla^11^, Heiralde Marck^12^, Giovanni Cinà^7^, Iacopo Vagliano^7^, Jan Pander^13^, Joanna E. Klopotowska^1,2,3^, Jurjen van der Schans^9^, Joris E. Lieverse^1,2^, Daniel Fernández-Llaneza^7^, Romy M. P. Vos^8^, Menno Maris^7^, Izak Yasrebi-de Kom^7^, Kitty J. Jager^7,14^, Leonora van Drop-Grandia^15^, Linda Dusseljee-Peute^1,2,4,5^, Luuk B. Hilbrands^16^, Marcel S.G. Kwa^17^, Marieke A. R. Bak^7^, Mariette van den Hoven^7^, Martijn G. Kersloot^18^, Nicolette F. de Keizer^7^, Otto R. Maarsingh^7^, Paul Blank^19^, Piet Heingraaf^20^, Renée de Wildt^21^, Ron Herings^7,22^, Ron J. Keizer^23^, Ronald Cornet^7^, Ruben Boyd^24^, Sebastiaan L. Knijnenburg^18^, Sipke Visser^25^, Stephanie Medlock^1,5,6^, Stijn Gremmen^26^, Teun van Gelder^27^, Tjerk S. Heijmens Visser^28^, Vianda S. Stel^7,29^.

^1^ Department of Medical Informatics, Amsterdam UMC location University of Amsterdam, Amsterdam, the Netherlands

^2^ Amsterdam Public Health, Digital Health, Amsterdam, The Netherlands

^3^ Amsterdam Public Health, Quality of Care, Amsterdam, The Netherlands

^4^ Amsterdam UMC Location University of Amsterdam, Department of Medical Informatics, eHealth Living & Learning Lab, Meibergdreef 9, Amsterdam, the Netherlands

^5^ Amsterdam Public Health, Aging and Later Life, Amsterdam, The Netherlands

^6^ Amsterdam Public Health, Methodology, Amsterdam, The Netherlands

^7^ Amsterdam University Medical Center

^8^ Vrije Universiteit Amsterdam

^9^ Open Universiteit

^10^ Nationale Intensive Care Evaluatie foundation

^11^ Amsterdam Economic Board

^12^ Koninklijke Nederlandse Maatschappij ter bevordering der Pharmacie [KNMP] Geneesmiddelen Informatie Centrum

^13^ AstraZeneca

^14^ European Renal Association-European Dialysis and Transplantation Association [ERA-EDTA] Registry

^15^ Z-Index

^16^ Radboud University Medical Center

^17^ College ter Beoordeling van Geneesmiddelen

^18^ Castor

^19^ NWO

^20^ Pitts.AI

^21^ Dutch Kidney Association

^22^ PHARMO Institute for Drug Outcomes Research

^23^ InsightRX

^24^ IXA

^25^ Digital Health Link

^26^ Dutch Kidney Foundation/Nierstichting

^27^ Leiden University Medical Center

^28^ CZ Health Insurance, Zorgverzekeraars Nederland

^29^ European Renal Association [ERA] Registry

## Abbreviations

ACE: Angiotensin-converting enzyme
AKI: Acute kidney injury
CDSS: Clinical decision support system
CKD: Chronic kidney disease
COREQ-32: Consolidated criteria for reporting qualitative health research
D-AKI: Drug-induced AKI
EGFR: Estimated glomerular filtration rate
GP: General practitioner
LEAPFROG: LEveraging real-world dAta to optimize PharmacotheRapy outcomes in multimOrbid patients by using machine learning and knowledGe representation methods
NSAIDS: Non-steroidal anti-inflammatory drugs
OTC: Over-the-counter
PPI: Proton pump inhibitor
SCR: Serum creatinine
TDM: Therapeutic drug monitoring

## References

[1] Susantitaphong P, Cruz DN, Cerda J, Abulfaraj M, Alqahtani F, Koulouridis I, et al. World incidence of AKI: a meta-analysis. Clin J Am Soc Nephrol. 2013;8(9):1482–93. 23744003 10.2215/CJN.00710113 PMC3805065

[2] Dasta JF, Kane-Gill S. Review of the Literature on the Costs Associated With Acute Kidney Injury. J Pharm Pract. 2019;32(3):292–302. 31291842 10.1177/0897190019852556

[3] Stottlemyer BA, Tran T, Suh K, Kane-Gill SL. A Systematic Review of the Costs of Drug-Associated Acute Kidney Injury and Potential Cost Savings With Nephrotoxin Stewardship Prevention Strategies. Clin Pharmacol Ther. 2025;117(4):989–1004. 39535321 10.1002/cpt.3493 PMC11924149

[4] Garcia G, Pacchini VR, Zamoner W, Balbi AL, Ponce D. Drug-induced acute kidney injury: a cohort study on incidence, identification of pathophysiological mechanisms, and prognostic factors. Front Med (Lausanne). 2024;11:1459170. 39534223 10.3389/fmed.2024.1459170 PMC11554514

[5] Hoste EA, Bagshaw SM, Bellomo R, Cely CM, Colman R, Cruz DN, et al. Epidemiology of acute kidney injury in critically ill patients: the multinational AKI-EPI study. Intensive Care Med. 2015;41(8):1411–23. 26162677 10.1007/s00134-015-3934-7

[6] Uchino S, Kellum JA, Bellomo R, Doig GS, Morimatsu H, Morgera S, et al. Acute Renal Failure in Critically Ill PatientsA Multinational, Multicenter Study. JAMA. 2005;294(7):813–8. 16106006 10.1001/jama.294.7.813

[7] Liu C, Yan S, Wang Y, Wang J, Fu X, Song H, et al. Drug-Induced Hospital-Acquired Acute Kidney Injury in China: A Multicenter Cross-Sectional Survey. Kidney Dis (Basel). 2021;7(2):143–55. 33824870 10.1159/000510455 PMC8010232

[8] Fernández-Llaneza D, Vos RMP, Lieverse JE, Gosselt HR, Kane-Gill SL, van Gelder T, et al. An Integrated Approach for Representing Knowledge on the Potential of Drugs to Cause Acute Kidney Injury. Drug Saf. 2025;48(1):43–58. 39327387 10.1007/s40264-024-01474-w PMC11711143

[9] Barreto EF, Gaggani AM, Hernandez BN, Amatullah N, Culley CM, Stottlemyer B, et al. The Acute Kidney Intervention and Pharmacotherapy (AKIP) List: Standardized List of Medications That Are Renally Eliminated and Nephrotoxic in the Acutely Ill. Ann Pharmacother. 2025;59(4):371–7. 39230007 10.1177/10600280241273191 PMC11871987

[10] Zoccali C, Blankestijn PJ, Bruchfeld A, Capasso G, Fliser D, Fouque D, et al. Children of a lesser god: exclusion of chronic kidney disease patients from clinical trials. Nephrol Dial Transpl. 2019;34(7):1112–4. 10.1093/ndt/gfz023 30815678

[11] Gagne JJ, Khan NF, Raj TS, Patel LR, Choudhry NK. Strength of evidence for labeled dosing recommendations in renal impairment. Clin Trials. 2017;14(2):219–21. 27780884 10.1177/1740774516673818

[12] Amatullah N, Stottlemyer BA, Zerfas I, Stevens C, Ozrazgat-Baslanti T, Bihorac A, et al. Challenges in Pharmacovigilance: Variability in the Criteria for Determining Drug-Associated Acute Kidney Injury in Retrospective, Observational Studies. Nephron. 2023;147(12):725–32. 37607496 10.1159/000531916 PMC10776175

[13] Khan S, Loi V, Rosner MH. Drug-Induced Kidney Injury in the Elderly. Drugs Aging. 2017;34(10):729–41. 28815461 10.1007/s40266-017-0484-4

[14] Tominey S, Timmins A, Lee R, Walsh TS, Lone NI. Community prescribing of potentially nephrotoxic drugs and risk of acute kidney injury requiring renal replacement therapy in critically ill adults: A national cohort study. J Intensive Care Soc. 2021;22(2):102–10. 34025749 10.1177/1751143719900099 PMC8120571

[15] Cox ZL, McCoy AB, Matheny ME, Bhave G, Peterson NB, Siew ED, et al. Adverse drug events during AKI and its recovery. Clin J Am Soc Nephrol. 2013;8(7):1070–8. 23539228 10.2215/CJN.11921112 PMC3700703

[16] van Blijderveen JC, Straus SM, de Ridder MA, Stricker BH, Sturkenboom MC, Verhamme KM. Adherence to renal function monitoring guidelines in patients starting antihypertensive therapy with diuretics and RAAS inhibitors: a retrospective cohort study. Drug Saf. 2014;37(5):369–77. 24748427 10.1007/s40264-014-0160-0

[17] Phipps DL, Morris RL, Blakeman T, Ashcroft DM. What is involved in medicines management across care boundaries? A qualitative study of healthcare practitioners’ experiences in the case of acute kidney injury. BMJ Open. 2017;7(1):e011765. 28100559 10.1136/bmjopen-2016-011765 PMC5253539

[18] Howarth M, Bhatt M, Benterud E, Wolska A, Minty E, Choi KY, et al. Development and initial implementation of electronic clinical decision supports for recognition and management of hospital-acquired acute kidney injury. BMC Med Inf Decis Mak. 2020;20(1):287. 10.1186/s12911-020-01303-x PMC7640650 33148237

[19] Sonoda A. A clinical decision support system promotes the appropriate use of drugs in hospitalized patients with kidney impairment. J Pharm Health Care Sci. 2025;11(1):26. 40181469 10.1186/s40780-025-00431-8 PMC11970011

[20] Martin M, Wilson FP. Utility of Electronic Medical Record Alerts to Prevent Drug Nephrotoxicity. Clin J Am Soc Nephrol. 2019;14(1):115–23. 29622668 10.2215/CJN.13841217 PMC6364537

[21] Haase A, Stracke S, Chenot JF, Weckmann G. Nephrologists’ perspectives on ambulatory care of patients with non-dialysis chronic kidney disease - A qualitative study. Health Soc Care Community. 2019;27(4):e438–48. 30945392 10.1111/hsc.12744

[22] Simmonds R, Evans J, Feder G, Blakeman T, Lasserson D, Murray E, et al. Understanding tensions and identifying clinician agreement on improvements to early-stage chronic kidney disease monitoring in primary care: a qualitative study. BMJ Open. 2016;6(3):e010337. 26988353 10.1136/bmjopen-2015-010337 PMC4800136

[23] Kanagasundaram NS, Bevan MT, Sims AJ, Heed A, Price DA, Sheerin NS. Computerized clinical decision support for the early recognition and management of acute kidney injury: a qualitative evaluation of end-user experience. Clin Kidney J. 2016;9(1):57–62. 26798462 10.1093/ckj/sfv130 PMC4720208

[24] Alanzi MA, Tully MP, Lewis PJ. Exploring the challenges faced by foundation doctors when prescribing high risk medicines safely during the on-call period: A qualitative study. Br J Clin Pharmacol. 2024;90(2):548–56. 37872107 10.1111/bcp.15928

[25] Tong A, Sainsbury P, Craig J. Consolidated criteria for reporting qualitative research (COREQ): a 32-item checklist for interviews and focus groups. Int J Qual Health Care. 2007;19(6):349–57. 17872937 10.1093/intqhc/mzm042

[26] PharmacoInformatics Lab - LEAPfROG project. Accessed May 27. 2025. [Available from: https://www.pharmacoinformaticslab.nl/en/leapfrog/

[27] Microsoft Teams. Accessed May 27. 2025.: https://www.microsoft.com/en-us/microsoft-teams/group-chat-software/

[28] Ryan GW, Bernard HR. Techniques to identify themes. Field methods. 2003;15(1):85–109.

[29] MAXQDA. Software for qualitative data analysis, 1989–2025, VERBI Software. Consult. Sozialforschung GmbH, Berlin, Germany.

[30] Elkhadragy N, Ifeachor AP, Diiulio JB, Arthur KJ, Weiner M, Militello LG, et al. Medication decision-making for patients with renal insufficiency in inpatient and outpatient care at a US Veterans Affairs Medical Centre: a qualitative, cognitive task analysis. BMJ Open. 2019;9(5):e027439. 31129589 10.1136/bmjopen-2018-027439 PMC6537985

[31] Khan LM, Al-Harthi SE, Osman AM, Sattar MA, Ali AS. Dilemmas of the causality assessment tools in the diagnosis of adverse drug reactions. Saudi Pharm J. 2016;24(4):485–93. 27330379 10.1016/j.jsps.2015.01.010 PMC4908100

[32] Naranjo CA, Busto U, Sellers EM, Sandor P, Ruiz I, Roberts EA, et al. A method for estimating the probability of adverse drug reactions. Clin Pharmacol Ther. 1981;30(2):239–45. 7249508 10.1038/clpt.1981.154

[33] Gallagher RM, Kirkham JJ, Mason JR, Bird KA, Williamson PR, Nunn AJ, et al. Development and inter-rater reliability of the Liverpool adverse drug reaction causality assessment tool. PLoS ONE. 2011;6(12):e28096. 22194808 10.1371/journal.pone.0028096 PMC3237416

[34] Kuhn GJ. Diagnostic errors. Acad Emerg Med. 2002;9(7):740–50. 12093717 10.1111/j.1553-2712.2002.tb02155.x

[35] Murphy RM, Dongelmans DA, Kom IY, Calixto I, Abu-Hanna A, Jager KJ, et al. Drug-related causes attributed to acute kidney injury and their documentation in intensive care patients. J Crit Care. 2023;75:154292. 36959015 10.1016/j.jcrc.2023.154292

[36] Yazdani S, Hosseinzadeh M, Hosseini F. Models of clinical reasoning with a focus on general practice: A critical review. J Adv Med Educ Prof. 2017;5(4):177–84. 28979912 PMC5611427

[37] Pauker SG, Kassirer JP. The threshold approach to clinical decision making. N Engl J Med. 1980;302(20):1109–17. 7366635 10.1056/NEJM198005153022003

[38] Griffin BR, Wendt L, Vaughan-Sarrazin M, Hounkponou H, Reisinger HS, Goldstein SL, et al. Nephrotoxin Exposure and Acute Kidney Injury in Adults. Clin J Am Soc Nephrol. 2023;18(2):163–72. 36754005 10.2215/CJN.0000000000000044 PMC10103278

[39] Kane-Gill SL. Kidney Damage Biomarkers: A Missing Piece of the Diagnostic and Management Puzzle for Acute Drug-Related Kidney Diseases. Pharmacotherapy. 2025;45(11):714–20. 41090380 10.1002/phar.70071

[40] Chaïbi K, Dreyfuss D, Gaudry S. Biomarkers and the prediction of acute kidney injury and renal replacement therapy initiation: a dream within a dream. Intensive Care Med. 2025. 10.1007/s00134-025-08254-9 41400678

[41] Sidorenkov G, Navis G. Safety of ACE inhibitor therapies in patients with chronic kidney disease. Expert Opin Drug Saf. 2014;13(10):1383–95. 25148900 10.1517/14740338.2014.951328

[42] Yamout H, Levin ML, Rosa RM, Myrie K, Westergaard S. Physician Prevention of Acute Kidney Injury. Am J Med. 2015;128(9):1001–6. 25912198 10.1016/j.amjmed.2015.04.017

[43] Brachemi S, Bollée G. Renal biopsy practice: What is the gold standard? World J Nephrol. 2014;3(4):287. 25374824 10.5527/wjn.v3.i4.287 PMC4220363

[44] Perazella MA. Diagnosing drug-induced AIN in the hospitalized patient: a challenge for the clinician. Clin Nephrol. 2014;81(6):381–8. 24691017 10.5414/CN108301 PMC4326856

[45] Xie Y, Bowe B, Li T, Xian H, Balasubramanian S, Al-Aly Z. Proton pump inhibitors and risk of incident CKD and progression to ESRD. J Am Soc Nephrol. 2016;27(10):3153–63. 27080976 10.1681/ASN.2015121377 PMC5042677

[46] Lazarus B, Chen Y, Wilson FP, Sang Y, Chang AR, Coresh J, et al. Proton pump inhibitor use and the risk of chronic kidney disease. JAMA Intern Med. 2016;176(2):238–46. 26752337 10.1001/jamainternmed.2015.7193 PMC4772730

[47] Perazella MA. Diagnostic Testing in AKI: Let’s Move the Field Forward. J Hosp Med. 2017;12(5):380–1. 28459911 10.12788/jhm.2735

[48] Strasma A, Kulkarni SA. Overuse of Urine Eosinophils in the Diagnosis of Acute Interstitial Nephritis: A Teachable Moment. JAMA Intern Med. 2019;179(8):1131–2. 31180467 10.1001/jamainternmed.2019.1755

[49] Karimzadeh I, Barreto EF, Kellum JA, Awdishu L, Murray PT, Ostermann M, et al. Moving toward a contemporary classification of drug-induced kidney disease. Crit Care. 2023;27(1):435. 37946280 10.1186/s13054-023-04720-2 PMC10633929

[50] Kwan JLLL, Sampson M, Shojania KG. Medication Reconciliation During Transitions of Care as a Patient Safety Strategy. Ann Intern Med. 2013;158(5Part2):397–403. 23460096 10.7326/0003-4819-158-5-201303051-00006

[51] Mastellos N, Car J, Majeed A, Aylin P. Using information to deliver safer care: a mixed-methods study exploring general practitioners’ information needs in North West London primary care. J Innov Health Inf. 2014;22(1):207–13. 10.14236/jhi.v22i1.77 25924550

[52] Rey A, Gras-Champel V, Choukroun G, Masmoudi K, Liabeuf S. Risk factors for and characteristics of community- and hospital-acquired drug-induced acute kidney injuries. Fundam Clin Pharmacol. 2022;36(4):750–61. 35037310 10.1111/fcp.12758 PMC9545588

[53] Polat EC, Koc A, Demirkan K. The role of the clinical pharmacist in the prevention of drug-induced acute kidney injury in the intensive care unit. J Clin Pharm Ther. 2022;47(12):2287–94. 36394173 10.1111/jcpt.13811

