# Supplementary material for: Hospital physicians’ reasoning and information needs in the diagnosis and prevention of drug-induced acute kidney injury: a qualitative study
Source: BMC Nephrol. 2026 Mar 4;27:231. doi: 10.1186/s12882-026-04852-x (PMC13067577; doi:10.1186/s12882-026-04852-x)
Supplement: Supplementary file 1 — Supplementary Material 1: Table S1: Interview guide. Table S2: Code tree [file 12882_2026_4852_MOESM1_ESM.doc]

***TABLE S1. Interview guide***

***General***

| Location |
| --- |
| Interviewer |
| Date |
| Start time |
| End time |

***Before start***

| **Interview explanation:**   - Informed consent - Q&A - Questions from participants - Notes will be taken - Audio (and video) recording - Duration + time limit for questions - Contact details of researchers - Focus on d-AKI and what it means - [Start recording:] Obtain informed consent once more |
| --- |

***Detection***

| **Main questions** | **Prompting questions** |
| --- | --- |
| **Question 1a.**  What information do you take into consideration to determine whether d-AKI is present? Walk me through your clinical reasoning process regarding this. (1)  **Question 1b.**  At what point do you consider a relationship between nephrotoxicity and a medication? Can you tell me more about this process? | **Question 1p1.**  Is this information available in the hospital's EHR, or is it found elsewhere? Do you need any other additional sources, and if so, which ones? (1)  **Question 1p2.**  Is there any specific information you would like to have but currently do not?  **Question 1p3.**  *If a specific situation/context is mentioned;* What about in other situations? Would you take the same approach in different cases? Why or why not? How would you handle it? |
| **Question 2.**  What do you think are the challenges in detecting d-AKI? (2) | **Question 2p1.**  What strategies do you currently use to detect d-AKI?  **Question 2p2.**  Do you remember a patient where d-AKI was not detected in time? What caused that? What were the circumstances?  **Question 2p3.**  What could help in detecting d-AKI at an earlier stage? |
| **Question 3.**  What could explain why nephrotoxic medications are not stopped or adjusted in time when kidney function deteriorates? (3) |  |
| **Question 4.**  How much kidney function loss, in eGFR, do you consider acceptable and anticipated when starting or continuing pharmacological therapy? At what point do you consider this unacceptable? (4) |  |

***Prevention***

| **Main questions** | **Prompting questions** |
| --- | --- |
| **Question 5.**  How do you balance adequate pharmacological therapy with protecting kidney function? Do you find this difficult at times? And if so, why? (5, 6) | **Question 5p1.**  What do you consider to be the most effective strategies for preventing d-AKI? (7, 8)  **Question 5p2.**  *If a specific situation/context is mentioned:* What about in other situations?  Would you take the same approach in different cases? Why or why not?  How would you handle it? |
| **Question 6.**  Is complete prevention of d-AKI achievable in your opinion? If yes, how? If no, why not? Explain what factors contribute to this. |  |

***End***

| **Main question** |
| --- |
| **Question 7.**  Is there anything you would like to add that hasn't been covered during this interview?  Question 8. |
| **Question 8.**  Who else should we speak with? |

1. Loghman-Adham M, Kiu Weber CI, Ciorciaro C, Mann J, Meier M. Detection and management of nephrotoxicity during drug development. Expert Opin Drug Saf. 2012;11(4):581-96.

2. Amatullah N, Stottlemyer BA, Zerfas I, Stevens C, Ozrazgat-Baslanti T, Bihorac A, et al. Challenges in Pharmacovigilance: Variability in the Criteria for Determining Drug-Associated Acute Kidney Injury in Retrospective, Observational Studies. Nephron. 2023;147(12):725-32.

3. Martin M, Wilson FP. Utility of Electronic Medical Record Alerts to Prevent Drug Nephrotoxicity. Clin J Am Soc Nephrol. 2019;14(1):115-23.

4. Chaumont M, Pourcelet A, van Nuffelen M, Racape J, Leeman M, Hougardy JM. Acute Kidney Injury in Elderly Patients With Chronic Kidney Disease: Do Angiotensin-Converting Enzyme Inhibitors Carry a Risk? J Clin Hypertens (Greenwich). 2016;18(6):514-21.

5. Haase A, Stracke S, Chenot JF, Weckmann G. Nephrologists' perspectives on ambulatory care of patients with non-dialysis chronic kidney disease - A qualitative study. Health Soc Care Community. 2019;27(4):e438-e48.

6. Wu H, Huang J. Drug-Induced Nephrotoxicity: Pathogenic Mechanisms, Biomarkers and Prevention Strategies. Curr Drug Metab. 2018;19(7):559-67.

7. James L, Wong G, Craig JC, Howard K, Howell M, Tong A. Nephrologists' perspectives on cancer screening in patients with chronic kidney disease: An interview study. Nephrology (Carlton). 2019;24(4):414-21.

8. Perazella MA. Renal vulnerability to drug toxicity. Clin J Am Soc Nephrol. 2009;4(7):1275-83.

***TABLE S2. Code tree***

| **Code System** | | | | | |
| --- | --- | --- | --- | --- | --- |
| Code System | | | | | |
|  | Prevention d-AKI | | | | |
|  |  | Determination elements | | | |
|  |  |  | Clinical symptoms | | |
|  |  |  |  | Limitation clinical symptoms | |
|  |  |  | Fluid balance list | | |
|  |  |  |  | Limitation fluid balance list | |
|  |  |  | (Time) relation drug - effect | | |
|  |  |  | Drug overview | | |
|  |  |  |  | Limitation drug overview | |
|  |  |  | Anamnesis | | |
|  |  |  | Rule out other causes | | |
|  |  |  | Echo | | |
|  |  |  |  | Limitation echo | |
|  |  |  | Laboratory | | |
|  |  |  |  | Limitation laboratory | |
|  |  |  | Biopsy | | |
|  |  |  |  | Limitation biopsy | |
|  |  |  |  |  | Mechanism determines damage |
|  |  |  | Patient characteristics | | |
|  |  |  |  | Limitation patient characteristics | |
|  |  | Risk assessment | | | |
|  |  |  | Risk factors d-AKI | | |
|  |  |  |  | Medical history | |
|  |  |  |  | Baseline kidney function low | |
|  |  |  |  | Low-risk patient | |
|  |  |  |  | Patient status - vulnerability | |
|  |  |  |  | Comorbidity | |
|  |  |  |  | Age | |
|  |  |  | High-risk patient | | |
|  |  |  |  | Additional burdensome monitoring | |
|  |  |  | Low-risk patient | | |
|  |  | Additional kidney function check | | | |
|  |  | Adequate hydration | | | |
|  |  | Physician knows medical history (sufficiently) | | | |
|  |  |  | Patient does (not) share medical history with doctor | | |
|  |  | Patient education | | | |
|  |  |  | Patient education is insufficient | | |
|  |  | Prescribing vigilance | | | |
|  |  |  | Avoid in vulnerable patient | | |
|  |  |  | Use safer alternative | | |
|  |  |  | Avoid and/or stop (prescribing) | | |
|  |  |  | Adjust for low kidney function | | |
|  |  | Existing support | | | |
|  |  |  | Pharmacy collaboration | | |
|  |  |  |  | Alert from pharmacy when kidney function is low, decreasing, or absent | |
|  | Determination d-AKI | | | | |
|  |  | Determination elements | | | |
|  |  |  | Clinical symptoms | | |
|  |  |  |  | Limitation clinical symptoms | |
|  |  |  | Fluid balance list | | |
|  |  |  |  | Limitation fluid balance list | |
|  |  |  | (Time) relation drug - effect | | |
|  |  |  | Drug overview | | |
|  |  |  |  | Limitation drug overview | |
|  |  |  | Anamnesis | | |
|  |  |  |  | Limitation anamnesis | |
|  |  |  | Rule out other causes | | |
|  |  |  | Echo | | |
|  |  |  |  | Limitation echo | |
|  |  |  | Laboratory | | |
|  |  |  |  | Limitation laboratory | |
|  |  |  | Biopsy | | |
|  |  |  |  | Limitation biopsy | |
|  |  |  |  |  | Mechanism determines damage |
|  |  |  | Patient characteristics | | |
|  |  |  |  | Limitation patient characteristics | |
|  |  | Risk assessment | | | |
|  |  |  | Risk factors d-AKI | | |
|  |  |  |  | Medical history | |
|  |  |  |  | Baseline kidney function low | |
|  |  |  |  | Low-risk patient | |
|  |  |  |  | Patient status - vulnerability | |
|  |  |  |  | Comorbidity | |
|  |  |  |  | Age | |
|  |  |  | High-risk patient | | |
|  |  |  |  | Additional burdensome monitoring | |
|  |  |  | Low-risk patient | | |
|  |  | Point in time | | | |
|  |  | Method | | | |
|  |  | Level of certainty | | | |
|  |  |  | Suspicion AKI | | |
|  |  |  | Confirmation AKI | | |
|  |  |  | Suspicion d-AKI | | |
|  |  |  | Confirmation d-AKI | | |
|  | Information needs | | | | |
|  |  | Dilemma | | | |
|  |  | Missing/unavailable/non-usable data | | | |
|  |  | Information not needed | | | |
|  |  | Information needed | | | |
|  | D-AKI treatment | | | | |
|  |  | Avoid and/or stop | | | |
|  |  | Improve the underlying problem and/or symptoms | | | |
|  |  | Administer antagonist | | | |
|  | Awareness and recognition of D-AKI | | | | |
|  |  | Awareness | | | |
|  |  |  | Barrier to awareness | | |
|  |  | Consciousness | | | |
|  | D-AKI occurs despite prevention | | | | |
|  |  | Reason: protocol not followed | | | |
|  |  | Reason: kidney function not monitored in time | | | |
|  |  | Reason: CDSS alert not followed up | | | |
|  |  | Reason: other information unknown | | | |
|  |  | Reason: current kidney function unknown | | | |
|  |  | Reason: acute situation and/or high urgency | | | |
|  |  | Reason: safer medication not available | | | |
|  |  | Reason: lack of attention | | | |
|  | CDSS | | | | |
|  |  | Proposed function | | | |
|  |  | Anti function | | | |
|  |  | Attitude | | | |
|  |  | Alert fatigue | | | |
|  |  | Differences between departments | | | |
|  | Type of d-AKI | | | | |
|  | Role | | | | |
|  | Missed cases | | | | |
|  | Drugs implicated in other side-effects (no d-AKI) | | | | |
|  | Drug implicated in d-AKI | | | | |
|  | Acceptable level of kidney function loss | | | | |
|  | Dilemma | | | | |
|  | Missing/unavailable/non-usable data | | | | |
|  | EHR | | | | |
|  | /// relevant | | | | |
|  | /// expression of vulnerability – personal feeling | | | | |
|  | /// risk of bias | | | | |
